# Social Media’s Use and Impact on Oral Surgeons and Oral Surgery Residents

**DOI:** 10.7759/cureus.31865

**Published:** 2022-11-24

**Authors:** Aurelien Godard

**Affiliations:** 1 Oral Surgery, Strasbourg University Hospital, Strasbourg, FRA

**Keywords:** social media applications, health professionnal education, social media communication, oral surgery, professionnal communication

## Abstract

Introduction: Social networks are now an effective tool in medical communication used in many specialties. It provides wide access to patients and pears communities because the engagement of all age ranges on Facebook and Instagram is growing. More and more training programs are using these platforms to promote themselves to future students, including those in oral and maxilla-facial surgery. But the impact of specialized medical content has never been studied on the population of working practitioners and residents.

Method: In this study, an anonymous online survey was distributed by email to French oral surgeons and French oral surgery residents. The questionnaire was accessible for a period of two months and included questions about demographic data and the professional use of different social networks.

Results: It received a total of 206 participations. All ages and professional statuses were represented. Around two-thirds of respondents were 25 to 35 years old. A large majority of the respondents (86%) had an account on at least one social media site, and 74% of those aged 25 to 35 were present on Facebook and Instagram at least. Residents at 65% affirmed they use social media as a source of medical information, in contrast to academic professors at 30%. This online content had a higher impact on residents’ medical experiences compared with other groups. Twenty-nine surgeons declared to post occasionally or regularly on social media; most of them were private practitioners. The role of social media in oral and maxilla-facial surgery practice was judged significant or inevitable by 44.3% of the respondents.

Conclusion: This preliminary survey of a small sample shows the growing interest of interns and young practitioners in medical content published by the professional community on social media as a source of non-institutional information.

## Introduction

The role of social media in our society is now unquestionable. Their daily uses for different purposes such as information, communication, or business between distant people have been integrated into the professional world. The medical community is becoming more and more permeable to new technologies. Professional office websites have become a basic activity for medical practitioners in order to increase their visibility and communicate with colleagues or potential future patients about their work [1].

In a survey performed in 2012 among plastic surgeon members of the American Society of Plastic Surgeons, Vardanian et al. demonstrated that 50% of the respondents used social media in their daily practice [2]. The same year, Haas and Park found that 55% of orthodontists who were members of the American Association of Orthodontists were using social media to increase their visibility [3]. Facebook was the predominant platform in both publications. The latter, available for the public since 2006, reported 2 billion active users monthly. Also, new platforms based on multimedia content were launched recently [4].

Meira et al. in 2021 showed higher Instagram use to search for a physician and practices in a sample of dentists, dental students, or patients [5]. The authors explained that it is a social media platform based mainly on visual content, which is very efficient for communicating with other practitioners and patients. Medical students are a growing social media target because of the health context related to COVID-19. Indeed, Yang et al. noticed a recent growth in the number of oral and maxillofacial surgery residency programs with an Instagram account. More than half (53%) were present on this platform in 2020 [6]. Saadeh et al. investigated medical and dental students at Jordan University, with 44% regularly using social media to inquire about medical information [7]. Butler et al. surveyed applicants to an orthopedic residency program and found that the most used platform to find information was Instagram [8].

So far, no study has been published that included a sample of oral and maxillofacial surgeons and investigated their opinions and use of widely available public social media.

The aim of this practice survey was to evaluate the impact and integration into medical practice of the most common social media platforms (Facebook, Twitter, and Instagram) among oral surgeons and residents and to collect their opinions on the impact of the contents consulted.

## Materials and methods

An anonymous survey made of 14 unique or multiple-choice questions has been designed thanks to the Google Forms tool (Google, Mountain View, U.S.A.). The online survey’s link (https://docs.google.com/forms/d/1xRNsyi-8FxuUtomfJn2KPb_8wf1I8LS-YDFYj2g7Lsc/edit) was sent by email to French oral surgeons and French oral surgery residents. All personal emails were acquired through publicly available data on the French Oral Surgery Society website or by direct request to the medical offices. Because of the diversity related to the practice of oral surgery, the practitioners questioned could be dental surgeons, stomatologists, or maxilla-facial surgeons. A local review board exemption was granted for this protocol. The consent of the participants was collected before sending the link to the online survey. A total of 1454 emails were sent. Two percent of the email addresses appeared to be false, and the email did not reach the addressee.

**Figure 1:**
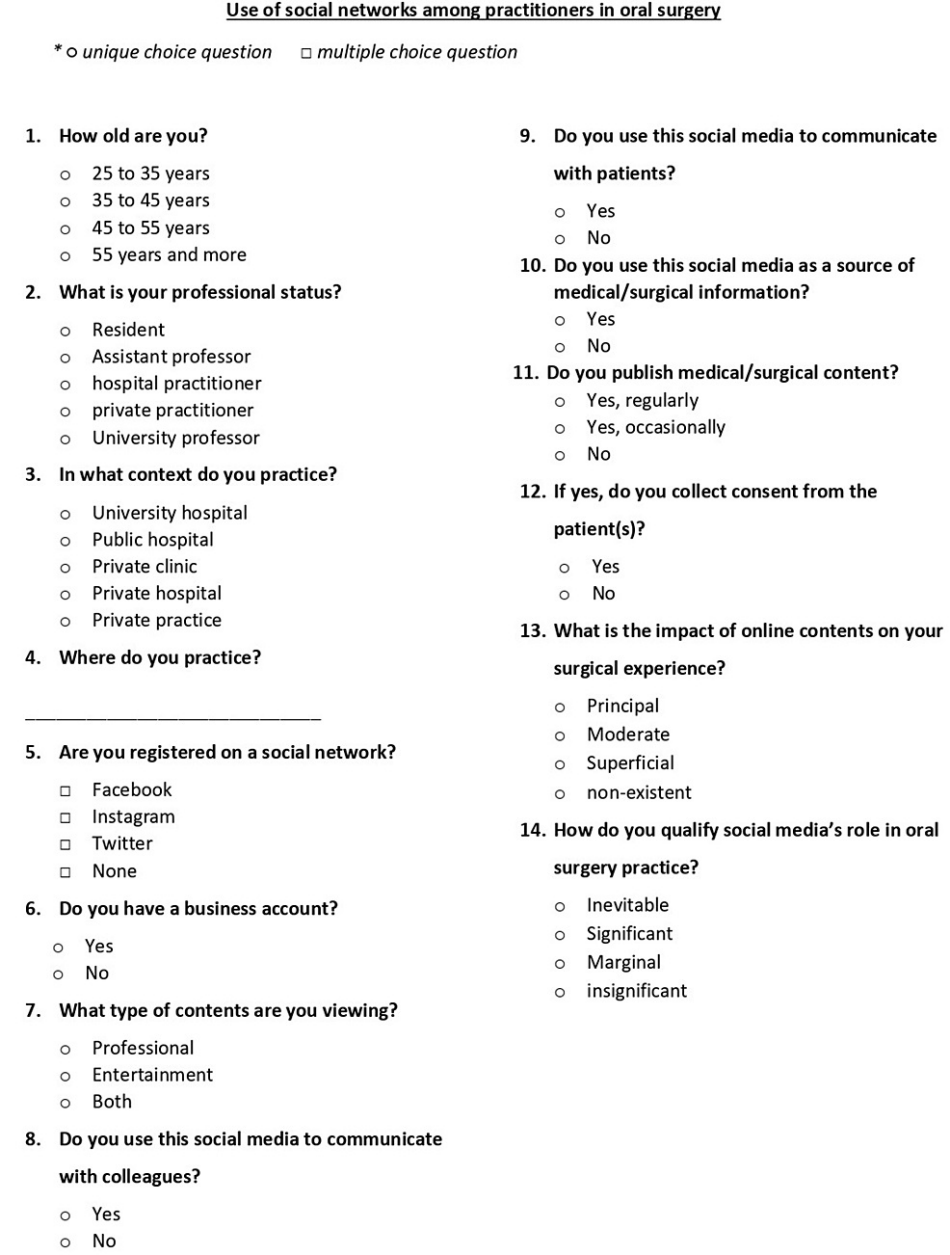
Copy of the online survey

First, questions focused on demographic data, including respondents’ age, professional status, practice location, and characteristics (private, public, academic, etc.). Then the survey assessed the surgeon’s adhesion to the main social media platforms, including Facebook, Twitter, and Instagram; the type of visited pages (entertainment or professional); and their own publication frequency. Finally, the participants were asked about their perception of the impact of these contents on their practice. The detailed questionnaire is available in Figure 1.

The study was launched in May 2021 and closed in July 2021, after which the data were compiled to be analyzed. The results were obtained and graphics were presented with Microsoft Excel software (Microsoft Corp., Redmond, Washington, U.S.A.).

## Results

**Figure 2:**
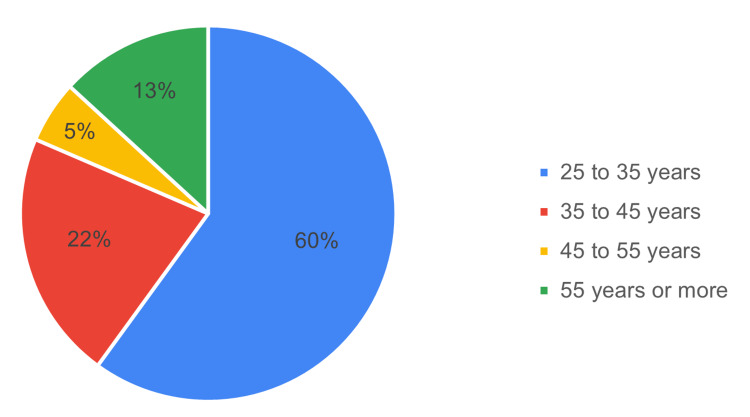
Respondent’s distribution in different age ranges

A total of two hundred and six practitioners completed the survey, which corresponded to a response rate of 14.4%. The age distribution is given in Figure 2. Sixty percent of the respondents were in the age range of 25-35 years. Ages 35 to 45 made up 21.5% of the total population.

One-quarter of the respondents were residents, and one-third were private practitioners. For those working in hospitals, 20.5% were assistant professors, 12.7% were practitioners without academic status, and 8.3% had academic status.

Regarding the practice characteristics in this sample, respondents declared 59% of them an activity in a university hospital, 58.3% in a private practice and/or clinic, and 17.5% in a public hospital. In this sample, only residents were able to have a partial or full private practice.

**Figure 3:**
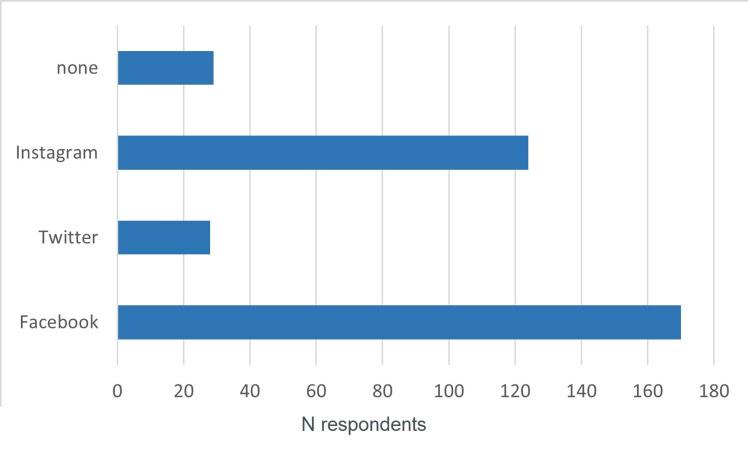
Surgeon’s engagement in different social medias

Paris and Provence-Alpes-Côte d’Azur were the areas with the most respondents (18.5%). Eighty-six percent of the respondents had an account on at least one social media. The one with the highest adhesion rate was Facebook, followed by Instagram. A small part of the participants did not engage in mentioning any social media. In practicians older than 55 years of age, it was noticed that 48% had at least one social media account. For the 25- to 35-year-old range, 74% of them were present on Facebook and Instagram at least. Figure 3 explains adhesion rates for each social media platform for all age ranges.

Eighty percent of the participants declared to consult professional and/or entertainment pages, while 11.7 % consult only professional pages. Forty-four percent of the participants answered positively to the question, "Do you use social media as a source of medical/surgical information?." Residents were 65% to consider social media as a source of medical or surgical information. This proportion decreased for the assistant professors and hospital practitioner groups, where they were 42% to confirm this setting. These publications were poorly considered by the academic professor's group, which presented a 70.6% negative answers rate.

**Figure 4:**
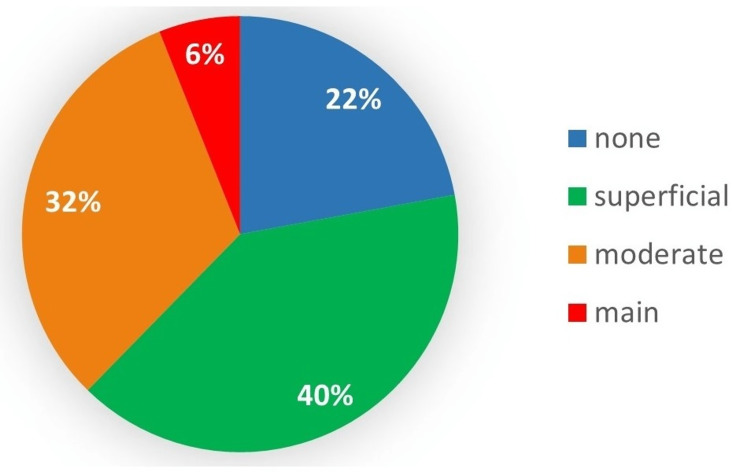
Perceived impact of social medias contents on surgeons’ experience

Figure 4 presents the answer distribution regarding the impact of online content on the surgical experience of participants; 38 of them reported a moderate or principal contribution to their surgical experience.

According to the answer distribution for each status, more than half (56%) of oral surgery residents reported moderate or significant participation in social media content during their surgical experience. None of the hospital practitioners with academic status reported that social media content was their primary source of information, while 13.3% stated a moderate contribution.

Regarding the utilization of social media, 43% of respondents declared to use social media to share with their peers and 9% to communicate with patients. Ten percent of the respondents reported occasional medical publications on social media, while 5% posted regular contributions. A total of 29 surgeons were occasional or regular contributors to one or more social media sites; 48.3% (n=14) of them belonged to the 25-35-year-old category, 37.3% (n=11) to the 35-45-year-old category, and four were older than 45 years. They practiced in the Paris area (24%), other countries (17%), and the Rhône-Alpes and Provence-Alpes-Côte-d'Azur districts (10% and 10%, respectively). Seventeen percent of surgeons who were engaged on social media had a professional-specific account.

Forty-three of the surgeons publishing on social media were private practitioners, followed by assistant professors (31%). It was noticed that 10% of the publications came from hospital practitioners or residents. Most of them used professional-specific accounts (64.3%).

**Figure 5:**
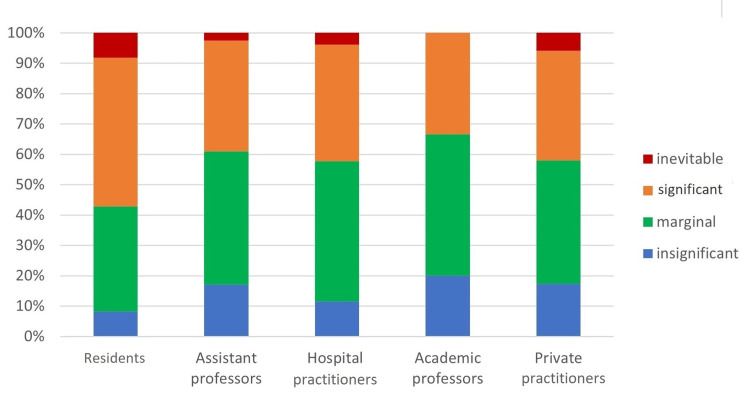
How do you qualify social media’s role in oral surgery practice? Answer's distribution for each professional status

The last question invited participants to qualify social media’s role in oral surgery practice. It was described as insignificant or marginal by 55.7% of respondents, whereas 44.3% considered that social media played a significant or inevitable role. Residents gave a higher appreciation as they were more than half (56%) to consider a significant or inevitable role, unlike hospital or private practitioners, who was only 42% gave this appreciation. One-third of academic professors judged that social media played a significant role in oral surgery. The proportion of responses for each occupational status is shown in Figure 5.

## Discussion

So far, no publication, including those by oral surgeons and residents studying social media's involvement in communication and formation, has been found.

In this study, a large amount of social media engagement can be observed in this sample of practitioners and residents, particularly with 82.5% of respondents present on Facebook and 60% on Instagram. Three-quarters of the 25- to 35-year-old respondents were present on these two platforms. Facebook was the dominant social media in several publications [2,3,9], but different authors also reported that Instagram is a rising platform, especially in younger age ranges, thanks to communication based mainly on photography [5-10].

Residents were more likely to consider social media content as a source of medical information (65%) than assistant professors or hospital practitioners (42%). We found the lowest rate of positive answers, with only 29%, in the academic professor’s group. Opposite results were found in the study published by Saadeh et al., in which 6.2% of surveyed students considered social media a trusted source of information [3]. The authors also indicated that using social media to inquire about medical information was decreasing with the number of completed years of exercise. This observation was confirmed by the present study, in which an assistant's or academic professor’s appreciation of medical information from social media was lower than that of residents. This social media content contributed more to the resident’s surgical experience than that of other groups. Both hospital and private practitioners declared the same level of participation in their surgical experiences.

Regarding social media utilization, most practitioners used it more to communicate with peers (43%), than with patients (9%). So far, most of the articles have investigated the surgeon’s communication with their future patients, but only a few numeric data were available. Vardanian et al. found that 27% of plastic surgeons mentioned "communicate with peers" as a reason to take part in social media usage [2]. Some other tools could also be utilized, like private inbox platforms or informal interactions.

A minority of the practitioners responding to the present survey used a professional-specific account, unlike what was described in previous studies in which medical professionals' use was performed with a specific account [3]. Management of specific personal and professional accounts for each social media platform may be difficult in daily utilization, which can explain why most of the respondents use unique accounts in this study.

Only 14% of these practitioners reported occasional or regular publishing activity. It is a major difference from similar published studies in which all surgeons using social media were considered regular contributors [2-11]. In this sample, private practitioners were more likely to post on social media. These data could be explained by the practice’s visibility related to the social media activity of the surgeon.

Less than half of this sample judged that social media took a significant or inevitable place in oral surgery practice. In 2012, Vardanian et al. explained that 56.7% of the surveyed plastic surgeons considered the involvement of social media as inevitable in surgery practice. It was an older sample, with an average age of 45 years or more. The highest rate of "significant" or "inevitable" answers was found in the 25-35-year-old group, which was predominant in the present survey. It seems that oral surgeons were less confident with social media involvement in their medical practice than plastic surgeons, but this appreciation is influenced by professional status because residents are more likely to consider that social media occupies a significant or inevitable place in surgical practice. Social media occupies a crucial place in social life, and the communication of young generations could increase medical students' and residents’ involvement in social media in their professional lives.

This survey received a response rate of 14% but involved only a small sample of 206 people. This sample cannot be considered representative of the population of practitioners and residents practicing oral surgery. Further research is needed to refine these results for the dental surgeon and oral and maxillofacial surgeon populations. Regarding the distinction between private and hospital or academic practice, a statement of bias exists because of the common oral surgeon’s dual private and public activity, except for residents and assistant professors.

The results showed a high social media engagement rate among the participants, especially on Facebook and Instagram, where they mostly used unique accounts. Respondents were more likely to communicate with peers than with patients. The majority of the respondents consulted professional pages, confirming the integration of medical content in current online activity. Residents reported that social media publications had a higher impact on their surgical experience than the other groups. However, more than one-third of private practitioners or assistant professors declared a moderate or important contribution. Social media provides a large diversity of contents that are not graded according to scientific pertinence, such as clinical photographs, which can be upgraded by different software.

Young practitioners and residents are more likely to be influenced by this content due to their limited clinical experience. This publication has to be treated carefully, and it has to be kept in mind that scientific literature remains the main standard of medical information sources. It could be easy to consider social media content as a scientific reference, but it should not replace evidence-based medicine supported by scientific publications from peer-reviewed journals.

New ethical considerations have been introduced with social media’s involvement in medics to patients communication as medical confidentiality and pictures property. Simplicio reported that the Brazilian Code of Ethics for Dentists had a specific chapter about public announcements, including online publications explaining the prohibition of pre-, per, or post-treatment photographs for commercial purposes and financial agreement information. Publishing every piece of information about a patient's identity was forbidden [12]. The French Medical Council advised practitioners to use internet platforms to inform about their practice conditions rather than direct commercial advertising. Medical information that is published should be honest and informative and should not expose unrealistic treatment outcomes [13]. Meira et al. demonstrated that orthodontists’ Instagram publications, especially before-and-after photographs, had a positive impact on practitioners’ credibility with patients and dental students [5].

Specific studies would be needed to appreciate the individual impact of each social media platform or to precise the most influential type of content on the medical or surgical experience of surgeons and residents. It would be relevant to investigate the sources of professional content consulted by the respondents. Indeed, medical content can come from official sources such as institutions or official scientific societies using social networks or from practitioners publishing on their own behalf. These contents could be prioritized according to their official or unofficial source. The credibility given to the different contents could be the objective of another investigation.

A practitioner's behavior on social networks is likely to influence how his/her peers judge his professionalism. Pronk et al. showed that the publication of unprofessional content by a practitioner had a negative influence on his or her perception by peers [14]. It is therefore clear that the image of practitioners on social networks contributes to their visibility and influence in their professional community. The American Medical Association reminds us that any content posted by a practitioner or student can have a negative impact on their reputation with their peers or patients and that they should regularly monitor their online presence [15].

There are several limitations to this study, including reporting bias, as it can be assumed that practitioners who were most supportive of social networking responded more widely to the survey. In addition, the proportion of respondents for each age group in this sample is not representative of the overall practitioner population. There are also disparities in the use and integration of different social networks, depending on the country. A wider sample, including practitioners not specialized in oral surgery in different countries, is necessary to increase the representativeness.

## Conclusions

This work provides an overview of medical oral surgeons' and residents’ opinions about the involvement of major social media in medical practice. We can see constant engagement in every age category and professional status. This increasing presence of practitioners on social media highlights new aspects of the healthcare economy, which tend to be a "facility" that is modifying the patient-doctor relationship, especially in surgical specialties such as facial aesthetic surgery or dental implantology.

The results of this study should be viewed with caution, but a greater impact of content posted on social networks by their peers on interns and young practitioners can be observed. These social networks could constitute a vector of information between practitioners in the absence of dedicated professional networks. One must remain cautious as there is no quality assessment of the published content. Peer networking could provide enhancement of medical experience sharing but new surgeons have to stay critical about social media pitfalls.
